# TurboID-EV: Proteomic Mapping of Recipient Cellular Proteins Proximal to Small Extracellular Vesicles

**DOI:** 10.1021/acs.analchem.3c01015

**Published:** 2023-09-14

**Authors:** Yuka Li, Eisuke Kanao, Tomoyoshi Yamano, Yasushi Ishihama, Koshi Imami

**Affiliations:** † Department of Molecular Systems BioAnalysis, Department of Proteomics and Drug Discovery, Graduate School of Pharmaceutical Sciences, Kyoto University, Kyoto 606-8501, Japan; ‡ Laboratory of Clinical and Analytical Chemistry, National Institute of Biomedical Innovation, Health and Nutrition, Osaka 567-0085, Japan; § Department of Immunology, Graduate School of Medical Sciences, Kanazawa University, Kanazawa 920-1164, Japan; ∥ WPI Nano Life Science Institute (NanoLSI), Kanazawa University, Kanazawa 920-1164, Japan; ⊥ PRESTO, Japan Science and Technology Agency (JST), Chiyoda-ku, Tokyo 102-0075, Japan; # Proteome Homeostasis Research Unit, RIKEN Center for Integrative Medical Sciences, Yokohama 230-0045, Japan; ¶ Department of Proteomics and Drug Discovery, Graduate School of Pharmaceutical Sciences, Kyoto University, Kyoto 606-8501, Japan

## Abstract

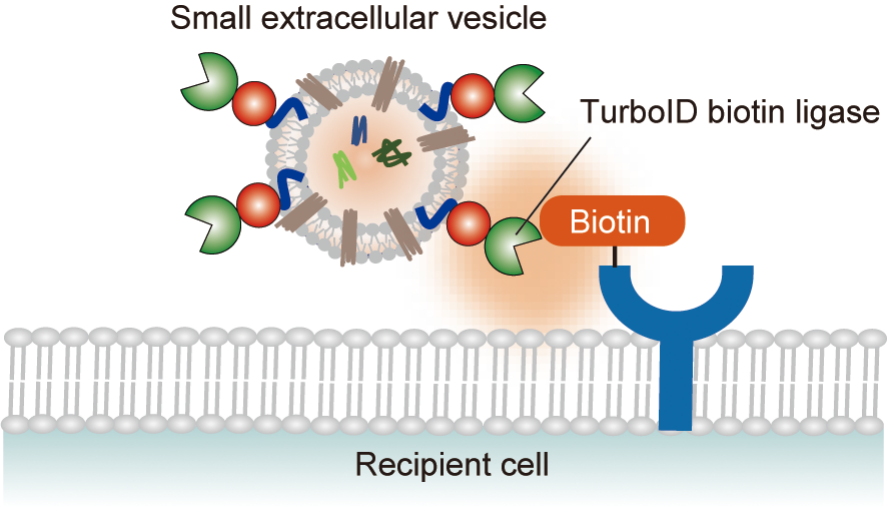

Extracellular vesicles (EVs), including exosomes, have been recognized as key mediators of intercellular communications through donor EV and recipient cell interaction. Until now, most studies have focused on the development of analytical tools to separate EVs and their applications for the molecular profiling of EV cargo. However, we lack a complete picture of the mechanism of EV uptake by the recipient cells. Here, we developed the TurboID-EV system with the engineered biotin ligase TurboID, tethered to the EV membrane, which allowed us to track the footprints of EVs during and after EV uptake by the proximity-dependent biotinylation of recipient cellular proteins. To analyze biotinylated recipient proteins from low amounts of input cells (corresponding to ∼10 μg of proteins), we developed an integrated proteomic workflow that combined stable isotope labeling with amino acids in cultured cells (SILAC), fluorescence-activated cell sorting, spintip-based streptavidin affinity purification, and mass spectrometry. Using this method, we successfully identified 456 biotinylated recipient proteins, including not only well-known proteins involved in endocytosis and macropinocytosis but also other membrane-associated proteins such as desmoplakin and junction plakoglobin. The TurboID-EV system should be readily applicable to various EV subtypes and recipient cell types, providing a promising tool to dissect the specificity of EV uptake mechanisms on a proteome-wide scale.

## Introduction

Extracellular vesicles (EVs) are lipid bilayer vesicles that contain various biomolecules such as proteins, DNA, and RNA.^1^ EVs are released from all cells and exhibit heterogeneity in size (40 to 1000 nm diameter) and in their cargo composition of biomolecules. In particular, small EVs (SEVs) of 50–200 nm diameter, including exosomes derived from late endosomal multivesicular bodies, have attracted a lot of attention due to their unique properties to enable intercellular communications.^2^ Although molecular profiles of SEV’s cargo RNAs, proteins, and lipids have been well-documented recently, we largely lack mechanistic insight into how SEVs are taken up by recipient cells and how the fate of SEVs is regulated within recipient cells after uptake.

Until now, SEV uptake is proposed to be mediated by receptor-mediated endocytosis, lipid rafts, phagocytosis, caveolae, macropinocytosis, and direct fusion at the plasma membrane to deliver the contents of SEVs into the cytoplasm of recipient cells.^3,4^ However, molecular mechanisms for SEV uptake differ between donor and recipient cell types. Such SEV- and cell-type specificity underlying SEV uptake appear to be regulated by the affinity between donor SEV proteins and recipient cellular proteins.^2^ For example, distinct exosomal integrin expression patterns are responsible for organ-specific uptake of exosomes through integrin-recipient cell surface protein interactions.^5,6^ Thus, a robust and versatile method to provide a proteomic landscape of EV-recipient protein interactions is required to dissect the diversity of the SEV uptake mechanisms. Although fluorescent-protein (e.g., GFP-CD63) tagged SEVs^7,8^ or high-speed atomic force microscopy^9^ was used to monitor SEV uptake, content delivery, and SEV-protein interaction within recipient cells, these methods do not provide a global view of proteins responsible for SEV uptake.

Recently, proximity-dependent biotinylation techniques have evolved in cell biology and biochemistry fields.^10^ The ability to biotinylate proximal proteins (∼10 nm apart) in combination with mass spectrometry (MS)-based proteomics is an attractive approach for profiling proteins in the proximity to SEVs. Indeed, proximity-labeling proteomics enabled the identification of EV surface and internal proteins using APEX2^11^ or wheat germ agglutinin conjugated to HRP^12^ as well as endosomal protein composition using a BioID system.^13^ However, the application of proximity labeling to elucidate SEV uptake mechanisms has been limited.

In this study, we developed an MS-based proteomic approach in which TurboID is fused to the SEV membrane, thereby facilitating the biotinylation of TurboID-EV proteins themselves and recipient cell proteins proximal to TurboID-EVs. By combining TurboID-EV biotinylation with stable isotope labeling with amino acids in cultured cells (SILAC),^14^ fluorescence-activated cell sorting (FACS), and spintip-based affinity purification of biotinylated proteins, we successfully identified proteins that are in close proximity to SEVs, potentially involved in their uptake and cellular interactions, related to clathrin-mediated endocytosis, macropinocytosis, and many other novel proteins.

## Methods

The experimental procedures are described in detail in the Supporting Information.

## Results and Discussion

### Overview of the TurboID-EV System

Among currently available biotin ligases and peroxidases, we chose TurboID because it has a higher biotinylation activity and higher temporal resolution (approximately >10 min) than other biotin ligases and does not require H_2_O_2_ treatment for biotinylation in contrast to peroxidases.^15^ We chose HEK293T as a model system because it exhibits high transfectability, allowing transient/stable expression of a gene of interest, and is widely used as a donor cell for SEVs due to its high SEV yield.^16^

**Figure 1:**
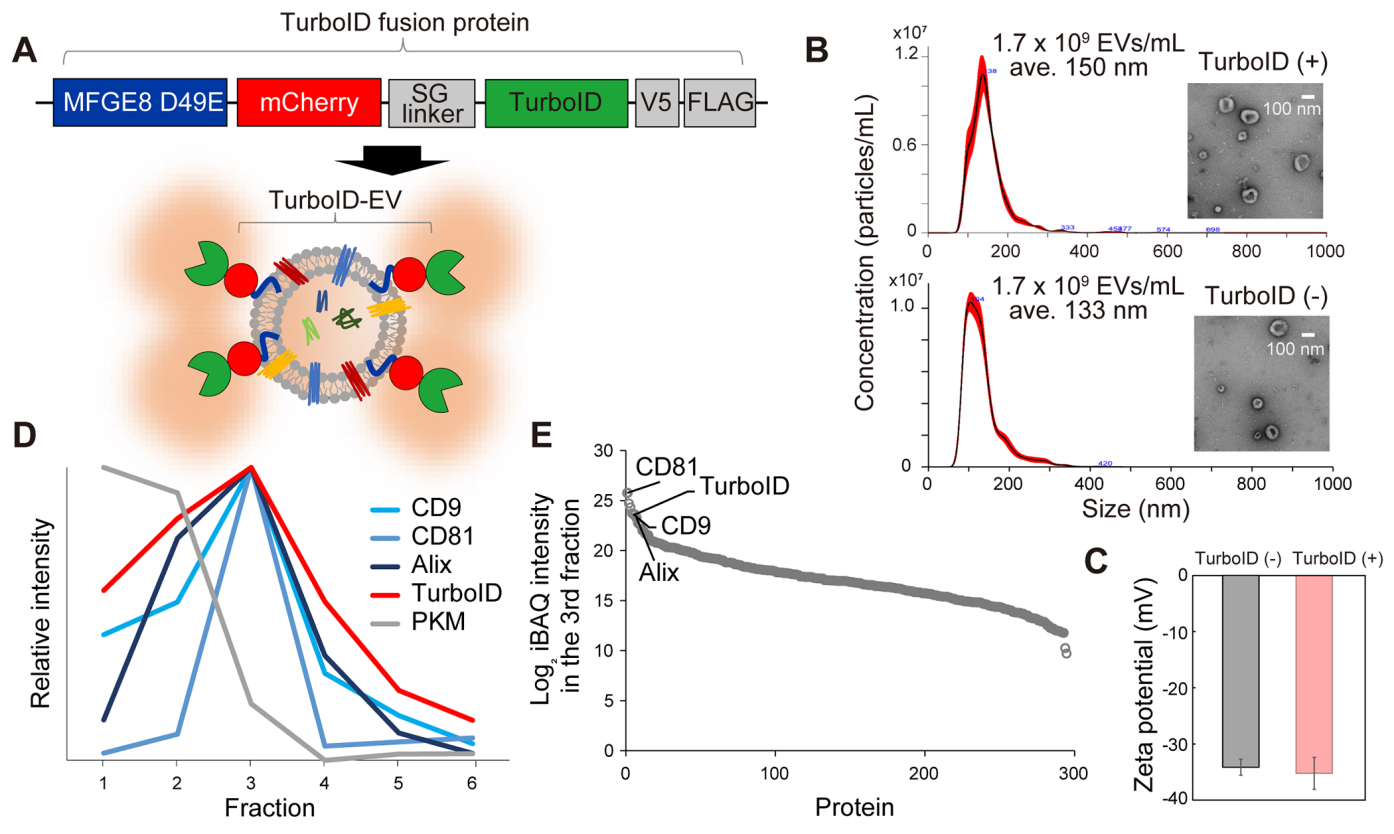
Evaluation of TurboID-EV production. (A) An overview of the TurboID fusion protein expression vector whose protein product is designed to be tethered to the SEV membrane. (B) Nanoparticle tracking analysis of SEVs collected from HEK293T cells expressing the TurboID proteins (top) and wild-type cells (bottom). The concentration and average diameter of SEVs from the TurboID-expressing cells were 1.7 × 10^9^ EVs/mL and 150 nm, respectively. For the wild-type cells, the concentration and average diameter of SEVs from the TurboID-expressing cells were 1.7 × 10^9^ EVs/mL and 133 nm, respectively. The inset shows transmission electron microscopy images of SEVs. (D) Relative protein abundance profiles of the TurboID fusion protein, selected SEV markers (CD9, CD81, and Alix), and a non-SEV protein (pyruvate kinase PKM) across iodixanol density gradient fractions. PKM is shown as an example that was not associated with SEVs based on its abundance profile. The “relative intensity” represents the signal intensity of each fraction divided by the highest intensity observed for a given protein. (E) Log_2_ iBAQ intensities of proteins quantified in the third fraction of the density gradient ultracentrifugation.

To identify EV interacting proteins through proximity-dependent biotinylation, we sought to design TurboID-EV, in which TurboID is tethered to the SEV membrane. To this end, we focused on phosphatidylserine (PS), one of the major constituent lipids of SEV membranes.^17^ The C1C2 domain of some lipid-related enzymes, including MFG-E8 (lactadherin), binds to PS^18,19^ and has been exploited as a SEV membrane anchor.^20^ Inspired by EV surface display technology using C1C2 domain,^21^ we generated a plasmid that expresses a fusion protein of TurboID with MFG-E8, mCherry, and epitope tags (Figure 1A). The arginine-glycine-aspartic acid (RGD) motif of MFG-E8 is known to promote the phagocytosis of apoptotic cells by binding to integrins expressed on macrophage cell membranes.^22^ To avoid this effect, we generated the TurboID fusion protein with a mutated version, MFG-E8 (D49E). The mCherry and epitope tags were introduced to visualize EVs in live cells and to detect the TurboID protein using immunoblotting, respectively. In summary, we developed an expression vector containing MFG-E8 D49E-mCherry-TurboID whose product is designed to be tethered to SEV lipid membranes, thereby enabling us to biotinylate proteins proximal to the TurboID-EVs.

### TurboID Fusion Protein Binds to Small EVs

To confirm the expression and biotinylation activity of the MFG-E8-mCherry-TurboID fusion protein, we first transfected a plasmid encoding the fusion protein into HEK293T cells. We observed mCherry-derived fluorescent dots in the cytoplasm (Figure S1A), similar to the localization pattern of MFG-E8 in HEK293T.^23^ We then tested and confirmed the biotinylation activity of the fusion proteins within cells by immunoblotting (Figure S1B) and by proteomic analysis based on nanoliquid chromatography/tandem mass spectrometry (LC/MS/MS) (Figure S1C and Table S1). We found that the TurboID fusion proteins biotinylated a broad spectrum of proteins in the cytosol, cell membrane, exosome, and endoplasmic reticulum (ER)-Golgi (Figure S1D). This result indicates that we observed multiple biotinylation events during the secretion of the TurboID proteins into the extracellular space through the ER-Golgi apparatus as well as the uptake of EVs containing the TurboID proteins into recipient cells.

We next assessed whether the TurboID fusion proteins secreted into the extracellular space bind to SEVs. We collected crude SEVs from cell culture media using ultracentrifugation (see Methods). Of note, no significant differences were observed in the properties of EVs, including their concentration, size, and zeta potential, between normal cells and TurboID-expressing cells (Figure 1B,C). This suggests that the expression of the TurboID proteins did not have a substantial effect on the overall characteristics of the EVs obtained in this study. To further distinguish SEVs from other particles, crude SEVs were separated by iodixanol-based density gradient ultracentrifugation, and 6 fractions from the top layer were sampled for quantitative proteomic analysis. We found that the TurboID fusion protein was coenriched in the third fraction with SEV marker proteins such as Alix (PDCD6IP), CD81, and CD9 (Figures 1D and S1E and Table S2), suggesting that the TurboID proteins were indeed associated with SEVs (hereafter referred to as “TurboID-EV”). We estimated the relative abundance of the 293 SEV proteins quantified in the third fraction using intensity-based absolute quantification (iBAQ^24^). iBAQ provides a rough estimation of relative protein abundance based on the sum of all of the peptide intensities divided by the number of theoretically observable peptides. The abundance of the TurboID fusion proteins was as high as that of the SEV markers (Figure 1E and Table S2), indicating that a reasonable number of the TurboID fusion proteins were successfully tethered to the SEVs.

### TurboID-EVs Can Be Uptaken by Recipient Cells

We next examined whether TurboID-EVs can be taken up by the recipient cells. A previous study performed a quantitative analysis of temporal EV uptake using HEK293T cells as a recipient model.^16^ Based on their findings, we selected 4 h as the coincubation time with EVs, as EV dots started to become observable at this time point and exhibited the highest number of EV dots within HEK293T cells.^16^ TurboID-EVs prepared using ultracentrifugation (Methods) were incubated for 4 h with normal HEK293T cells that did not express the TurboID proteins. Consistently, our results showed that the 4 h time point exhibited the highest number of EV dots (Figure S2A,B), confirming the uptake of TurboID-EVs. TurboID-EVs, thus, can serve as a valuable tool for monitoring EV uptake and fate within recipient cells.

### TurboID-EVs Retain Enzymatic Activity *in Vitro* and Can Biotinylate EV Proteins

**Figure 2:**
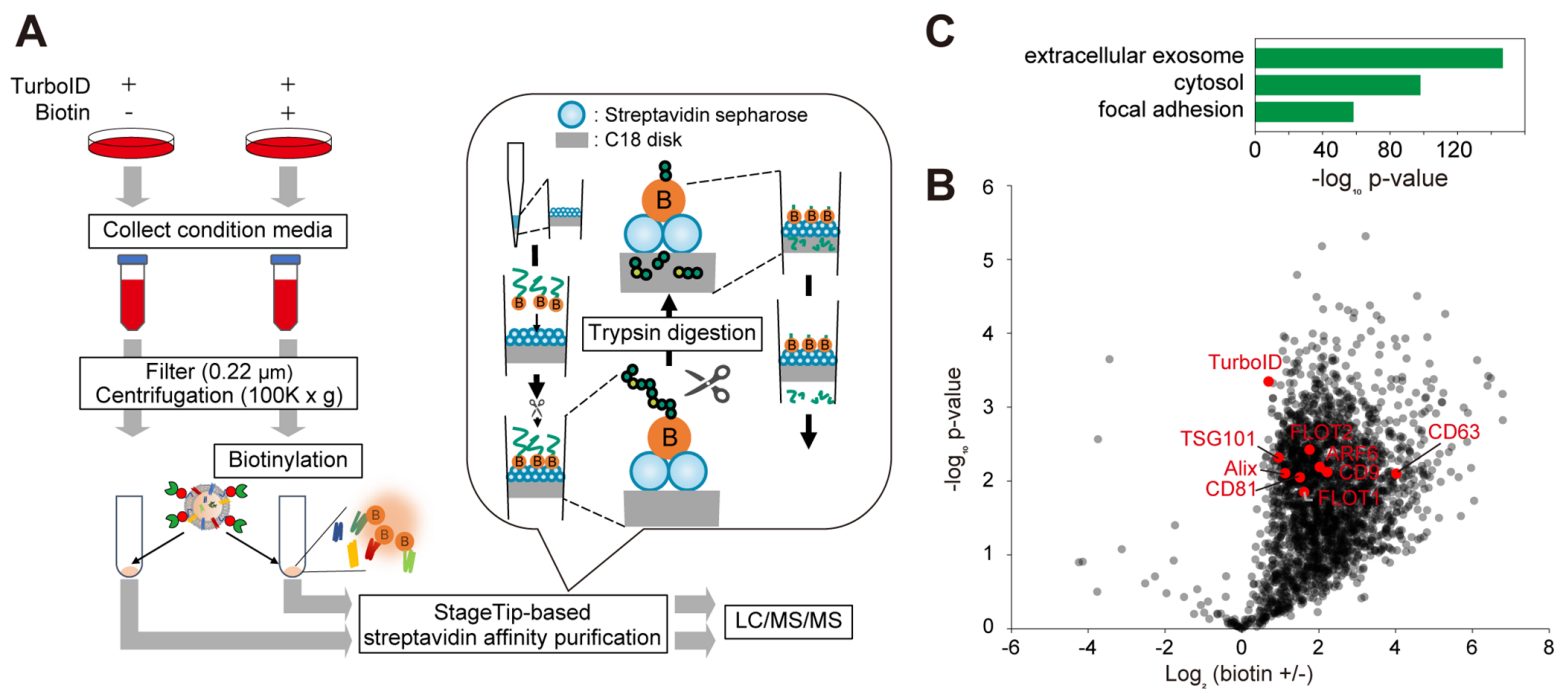
*In vitro* biotinylation of TurboID-EVs. (A) Experimental design for *in vitro* biotinylation of TurboID-EVs collected with ultracentrifugation. Biotinylated proteins were enriched with StageTips containing C18 disk and streptavidin sepharose. (B) A volcano plot showing differential biotinylation levels of proteins with or without biotin addition in TurboID-EV fractions. Three independent experiments were performed. (C) GO enrichment analysis of the significantly enriched proteins in the biotin (+) experiments (log_2_ fold-change > 1, *p* < 0.01). Only the top 3 GO cellular component terms are shown.

To confirm if TurboID-EVs are active after collecting them with ultracentrifugation, we performed *in vitro* biotinylation of EV proteins by incubating TurboID-EVs with exogenously added ATP and biotin (see Methods). As a control, an experiment without biotin addition was also done. Protein amounts for EV pellets after ultracentrifugation were approximately 10 μg, which was a much lower input for streptavidin-based affinity purification compared to a typical proximity labeling experiment where mg amounts of proteins are used.^10^ To efficiently enrich biotinylated proteins from a low input sample, we adapted and modified the fully integrated spintip-based affinity purification-MS technology (FISAP) method,^25^ which uses streptavidin sepharose in a StageTip^26^ for capturing and digesting biotinylated proteins (see Methods). Using FISAP and LC/MS/MS, we quantified 2,327 biotinylated proteins, of which 918 proteins were enriched in the biotin (+) samples (log_2_ fold-change > 1, *p* < 0.01) (Figure 2B and Table S3). Notably, gene ontology (GO) enrichment analysis using Database for Annotation, Visualization and Integrated Discovery (DAVID)^27^ revealed that exosome-related proteins, including CD9 and CD63, were highly enriched (Figure 2C). These results indicate that TurboID-EVs retained their activity and could biotinylate EV proteins, providing information about the protein composition of TurboID-EVs.

### TurboID-EVs Can Biotinylate Recipient Cellular Proteins

**Figure 3:**
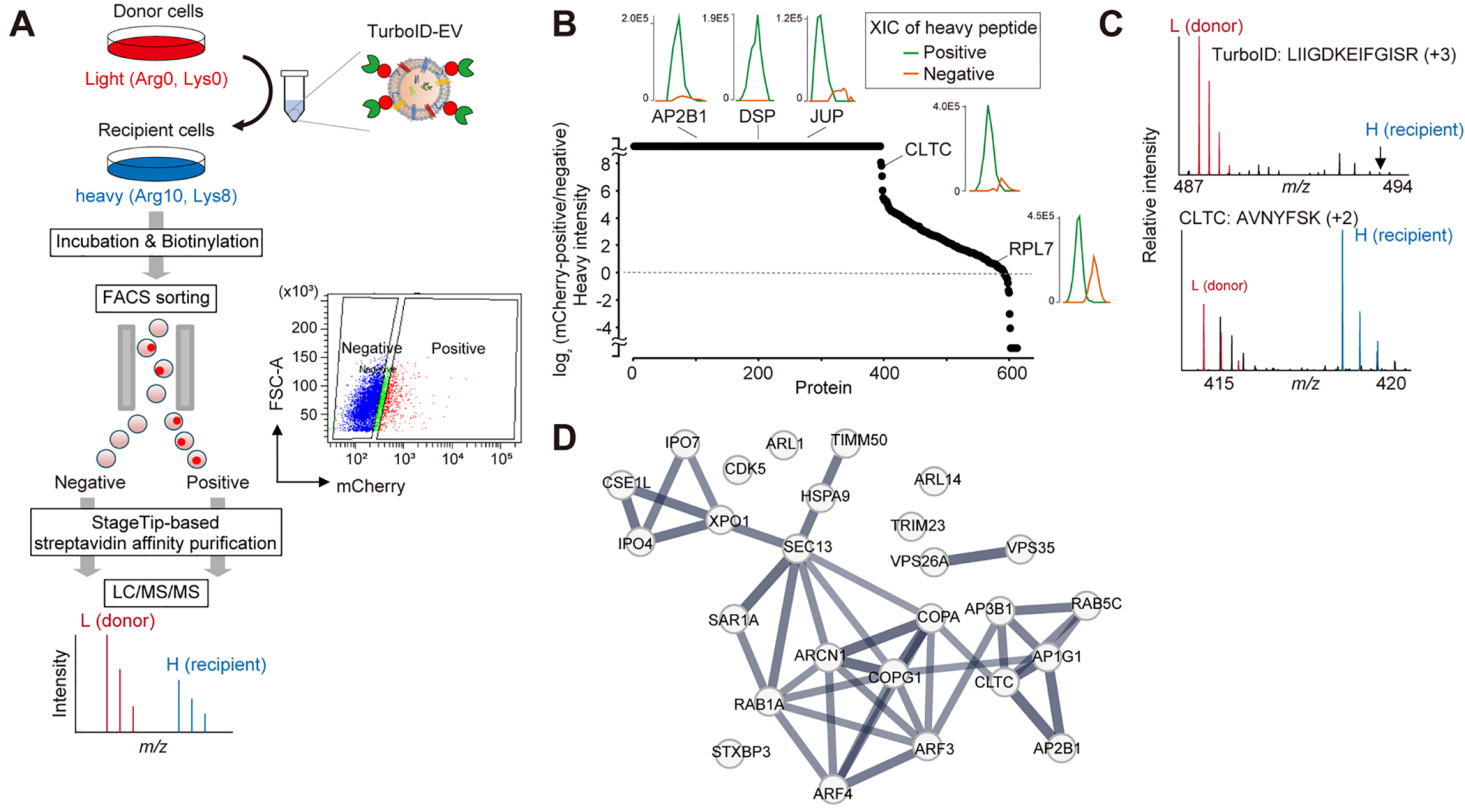
Identification of proteins in recipient cells proximal to TurboID-EVs during and after uptake. (A) Experimental design for identification of proteins in recipient HEK293T cells in close proximity to TurboID-EVs. The recipient cells were SILAC heavy-labeled. Only heavy-labeled proteins were monitored to identify and distinguish recipient proteins and compared between the mCherry-positive and -negative cells. (B) Log_2_ fold-change of the heavy-labeled recipient proteins between the mCherry-positive and -negative cells. Representative extracted ion chromatograms (XICs) of monoisotopic peaks of the heavy peptides of the selected proteins are shown for the mCherry-positive (green) and -negative (orange) cells. AP2B1: LASQANIAQVLAELK; DSP: VQYDLQK; JUP: LAEPSQLLK; CLTC: TSIDAYDNFDNISLAQR; RPL7: EVPAVPETLK (C) Exemplary MS spectra for TurboID (LIIGDKEIFGISR) (top) and CLTC (AVNYFSK) (bottom). (D) A STRING protein interaction network of the selected proteins involved in intracellular protein transport (based on the high-confidence network, >0.7).

Having confirmed the properties of TurboID-EVs, we finally applied the method to identify recipient cellular proteins proximal to TurboID-EVs during and after uptake. The TurboID fusion proteins should biotinylate both TurboID-EV proteins (donor; see Figure 2) and recipient cellular proteins. To distinguish whether biotinylated proteins are derived from donor EVs or recipient cells, we labeled recipient cells with heavy amino acids (Arg10, Lys8) based on SILAC,^14^ while donor TurboID-EVs were collected from unlabeled (Arg0, Lys0) cells (Figure 3A). We then treated and incubated the heavy-labeled recipient cells with TurboID-EVs and biotin for 4 h (see Methods). We observed that TurboID-EVs were taken up by recipient cells (Figures S2B and 3A), but only a few percent of the total cell population exhibited mCherry-positive signals (Figure 3A), consistent with a recent report showing that EV uptake is a process with low yields.^8^ This indicates that a highly sensitive method is required to analyze a low amount of biotinylated proteins from a few mCherry-positive cells. Indeed, we could not even identify the TurboID fusion protein in recipient cells with a conventional streptavidin affinity purification-MS workflow. We, therefore, sought to enrich low abundance biotinylated proteins by combining FACS sorting of mCherry-positive cells and the spintip-based enrichment of low-abundance biotinylated proteins (Figure 3A). Approximately 7.8 × 10^4^ mCherry-positive cells (∼10 μg protein) were sorted, and then, after spintip-based enrichment and digestion of biotinylated proteins, LC/MS/MS was used to analyze the resulting peptides. In parallel, we analyzed the mCherry-negative cells (7.8 × 10^4^ cells) from the same cell population as the control (Figures 3A and S2C).

This approach led to the quantification of 613 heavy-labeled proteins (i.e., recipient cellular proteins), of which 456 proteins (74%) exhibited at least 10-fold enrichment in the mCherry-positive cells relative to the negative cells (Figure 3B and Table S4), since only mCherry-positive cells are expected to be biotinylated by TurboID-EVs. The extracted ion chromatograms of the selected proteins also ensured reliable quantification (Figure 3B). Mass spectra exemplifying this are shown for the TurboID protein and the clathrin heavy chain 1 (CLTC) (Figure 3C); a peptide (LIIGDKEIFGISR) derived from the TurboID was exclusively observed as a light form, validating that TurboID-EV proteins from donor cells were unlabeled and not cross-labeled with heavy amino acids. In contrast, both light and heavy forms of CLTC peptides (e.g., AVNYFSK) were observed, consistent with the observations that CLTC is one of the SEV proteins^28^ and it is also involved in EV uptake.^29^

Endocytosis is one of the well-known mechanisms for the uptake of fine particles including viruses, EVs, and nanoparticles.^29,30^ Indeed, our approach identified proteins involved in clathrin-dependent endocytosis (e.g., CLTC, AP-2 complex subunit beta (AP2B1), AP-1 complex subunit gamma-1 (AP1G1), AP-3 complex subunit beta-1 (AP3B1), ras-related protein RAB5, and RAB7 and macropinocytosis-related proteins (e.g., filamin-A (FLNA), Coronin-1C (CORO1C), actin-related protein 2/3 complex subunit 2 (ARPC2), alpha-actinins (ACTN1/3/4), and fascin (FSCN1)). In addition to these, proteins involved in intracellular transport during and after EV uptake were identified and formed a highly connected protein interaction network based on the STRING database^31^ (Figure 3D). These results suggest that the TurboID-EV system could capture proteins potentially involved in a series of EV uptake processes. Importantly, we identified plasma membrane-associated proteins such as junction plakoglobin (JUP) and desmoplakin (DSP) related to the tight junction. These proteins might be newly identified key factors underlying SEV uptake, and further experiments, such as investigating the effect of loss-of-gene function with an SEV uptake assay, will be needed to validate their role in SEV uptake. Collectively, we demonstrated that the TurboID-EV system enabled us to identify recipient cellular proteins proximal to SEVs, which would provide an opportunity for tracking the footprints of EVs during and after their uptake.

## Conclusions

Here, we established the TurboID-EV system, which enables proximity-dependent biotinylation of proteins neighboring TurboID-EVs *in vitro* and in a cell culture system. While we demonstrated the utility of TurboID-EV using HEK293T cells as a model, this method should be readily applicable to different subtypes of TurboID-EVs (e.g., CD63-positive EVs or EV surface glycan structures^32^) as a donor EV and various cell types as acceptor cells, which should provide insights into the selectivity of EV uptake mechanisms. Despite the unique features of the TurboID-EV system, one limitation of the method is that donor cells need to express the exogenous TurboID fusion protein. This may limit the usability of the method to specific cell types and alter the physiological properties of EVs. It is also important to keep in mind that proximity-dependent biotinylation is known to label proteins, irrespective of whether they directly interact with the target proteins or are indirectly associated with them. Therefore, the proteins identified in this study are likely to include “bystander” proteins that are associated with EV-interacting proteins. Nevertheless, we demonstrated that the TurboID-EV system coupled with MS-based proteomics is a promising tool to reveal EV uptake mechanisms on a proteome-wide scale, beyond the limited scope of methods relying solely on fluorescent tagging or microscopy techniques.

## Data Availability

The proteomics data have been deposited to the ProteomeXchange Consortium via the jPOST^33^ partner repository (https://jpostdb.org) with the data set identifier PXD040569.

## Supporting Information Available

Table S1: Proximity-dependent proteomic analysis of HEK293T cells expressing the TurboID fusion proteins (related to Figure S1C); Table S2: Proteomic analysis of SEV proteins across the density gradient (related to Figure 1D,E); Table S3: Proximity-dependent proteomic analysis of TurboID-EVs (related to Figure 2B); Table S4: Proximity-dependent proteomic analysis of TurboID-EV-recipient cell interactions (related to Figure 3B) (ZIP)
Additional experimental details, materials, and methods (PDF)
