# Supplementary material for: TurboID-EV: Proteomic Mapping of Recipient Cellular Proteins Proximal to Small Extracellular Vesicles
Source: Anal Chem. 2023 Sep 14;95(38):14159–64. doi: 10.1021/acs.analchem.3c01015 (PMC10534987; doi:10.1021/acs.analchem.3c01015)
Supplement: Supplementary file 2 — ac3c01015_si_002.pdf [file ac3c01015_si_002.pdf]

Supporting Information

TurboID-EV: proteomic mapping of recipient cellular proteins proximal to small extracellular vesicles

Yuka Li [1], Eisuke Kanao [1,2], Tomoyoshi Yamano [3,4,5], Yasushi Ishihama* [1,2], Koshi Imami* [1,5,6]

[1] Department of Molecular Systems BioAnalysis, Graduate School of Pharmaceutical Sciences, Kyoto University, 606-8501 Kyoto, Japan
[2] Laboratory of Clinical and Analytical Chemistry, National Institute of Biomedical Innovation, Health and Nutrition, 567-0085 Osaka, Japan
[3] Department of Immunology, Graduate School of Medical Sciences, Kanazawa University, Kanazawa, Japan.
[4] WPI Nano Life Science Institute (NanoLSI), Kanazawa University, Kanazawa, Japan
[5] PRESTO, Japan Science and Technology Agency (JST), Chiyoda-ku, Tokyo 102-0075, Japan
[6] RIKEN Center for Integrative Medical Sciences, 230-0045 Yokohama, Japan

*Corresponding authors:
Koshi Imami
RIKEN Center for Integrative Medical Sciences,
Tel.: +81-45-503-9696
email: koshi.imami@gmail.com

Yasushi Ishihama
Graduate School of Pharmaceutical Sciences, Kyoto University
Tel.: +81-75-753-4555
Fax.: +81-75-753-4601
email: yishiham@pharm.kyoto-u.ac.jp

Table of Contents

1. Supplementary Experimental Page

1.1 Cloning …3
1.2 Cell culture …3
1.3 Transfection of plasmids …3
1.4 Ultracentrifugation for SEV isolation …3
1.5 Iodixanol density gradient fractionation (related to Figure 1D) …3
1.6 TurboID reaction in cells transiently expressing the TurboID proteins (related to Figure S1B) …4
1.7 TurboID reaction *in vitro* (related to Figure 2) …4
1.8 TurboID reaction in recipient cells (related to Figure 3) …5
1.9 Transmission electron microscopy (related to Figure 1B) …5
1.10 Nanoparticle tracking analysis (related to Figure 1B) …5
1.11 Immunoblotting (related to Figure S1B) …6
1.12 Zeta potential measurement (related to Figure 1C) …6
1.13 LC/MS/MS analysis …6
1.14 Proteomics data processing and analysis …7

2. Supplementary Figures

Figure S1: Mass spectrometric analysis of the TurboID fusion protein (related to Figure 1) …8
Figure S2: Evaluation of TurboID-EV uptake and FACS experiments (related to Figure 3A). …9

3. Supplementary References …10

1. Supplementary Experimental

1.1 Cloning

The entry clone pENTR221 MFG-E8 for Gateway cloning was purchased from DNAFORM, Japan. Site-directed mutagenesis was performed to create the D49E MFG-E8 mutant using a QuikChange Lightning site-directed mutagenesis kit (Agilent, USA) with the following primers; forward GTACGAGGGGAAGACCTCTCCTCGCACTTCT and reverse AGAAGTGCGAGGAGAGGTCTTCCCCTCGTAC. The MFG-E8 D49E coding DNA was then subcloned into the destination vector pDEST-pcDNA5 containing mCherry-TurboID with LR clonase 2 (Thermo Fisher Scientific, USA) using the Gateway system. All coding regions generated in this study were confirmed by Sanger dideoxynucleotide sequencing.

1.2 Cell culture

HEK293T cells (obtained from RIKEN BRC) were cultured in Dulbecco's Modified Eagle Medium (DMEM, Thermo Fisher Scientific) containing 10 % fetal bovine serum (FBS, Thermo Fisher Scientific) at 37 °C with 5 % CO_2. For SILAC labeling (related to Figure 3), HEK293T cells were cultured in arginine- and lysine-free DMEM (Thermo Fisher Scientific) supplemented with 10 % SEV-depleted FBS and heavy amino acids [0.398 mM L-(13C_6,$^{15}N_4$)-arginine (Arg“10”) and 0.798 mM L-($^{13}C_6$,$^{15}N_2$)-lysine (Lys“8”)] (Cambridge Isotope Laboratories, USA) for at least five doubling times. To prepare SEV-depleted FBS, SEVs in FBS were removed by ultracentrifugation at 100,000 G for 18.5 h, and supernatants were used as the SEV-depleted FBS.

1.3 Transfection of plasmids

For transfection of MFG-E8 D49E-mCherry-TurboID plasmid DNA into HEK293T cells, cells were cultured in a 15 cm dish to 50 % confluency on the day of the experiment and transfected with a mixture of 15 μg of the plasmid and 45 μL of 1.0 mg/mL polyethylenimine (Polysciences, USA), and then incubated for 24 h in a CO_2 incubator.

1.4 Ultracentrifugation for SEV isolation (related to Figures 1, 2, 3)

The procedure for purifying crude SEV pellets with ultracentrifugation was adapted from a standard protocol for SEV research [1] with a slight modification. Cell-conditioned medium was collected from 15 cm dishes of HEK293T cells at 80 % cell density. All of the following centrifugation steps were performed at 4 °C. Cells were further removed by centrifugation at 400 G for 10 min. Cell debris was then removed by centrifugation at 2,000 G for 10 min. To pellet and remove large EVs, the supernatant was ultracentrifuged at 15,000 G for 40 min. The supernatants from the 15,000 G step were passed through a 0.22 μm pore PES filter (Millipore), followed by ultracentrifugation at 120,000 G for 90 min (related to Figure 1G, 1H) or 100,000 G for 120 min (related to Figures 2, 3). The pellet was then washed with ice-cold PBS and ultracentrifuged at 120,000 G for 90 min or 100,000 G for 120 min to pellet SEVs.

1.5 Iodixanol density gradient fractionation (related to Figure 1D)

60 % (w/v) iodixanol (OptiPrep^{TM}) density medium (Serumwerk Bernburg AG, Germany) was diluted with ice-cold PBS to prepare 12-36 % gradients. Crude SEVs prepared according to 1.3 Ultracentrifugation for SEV isolation were added to the bottom of a centrifugation tube. The iodixanol solutions were layered on top and ultracentrifuged at 120,000 G for 16 h at 4 °C. Six fractions of 1 mL were collected from the top of the gradient. To precipitate proteins in individual fractions, trichloroacetic acid (TCA) was added to a final concentration of 20 % and incubated for 1 h on ice. Proteins were precipitated by centrifugation at 16,000 G for 30 min

at 4 °C. After the supernatant was removed, protein pellets were washed with 1 mL of ice-cold acetone, and centrifuged at 16,000 G for 5 min at 4 °C. This washing step was repeated three times, and the remaining acetone was evaporated with a centrifugal vacuum concentrator. The protein pellets were solubilized with 8 M urea in 0.1 M Tris-HCl pH 8.0. Reduction of cysteine residues was performed with 10 mM dithiothreitol (DTT) at room temperature for 30 min, followed by alkylation with 50 mM iodoacetamide (IAA) at room temperature for 30 min. The proteins were digested with 0.5 µg of LysC (Fujifilm Wako, Japan) and 0.25 µg of trypsin (Promega, USA) at 37 °C overnight. Peptides were acidified with 0.5 % TFA and desalted by C18-SCX and SDB-XC StageTips (Adachi et al. 2016)[2].

1.6 TurboID reaction in cells transiently expressing the TurboID proteins (related to S1B)

Biotinylation: 24 h after transfection with the MFG-E8 D49E-mCherry-TurboID plasmid, HEK293T cells were incubated and biotinylated with 50 µM biotin (1 mM stock in DMEM) for 30 min, while the biotin addition was omitted for control cells that expressed the TurboID fusion protein. Collected cell pellets were lysed with 1 mL RIPA buffer (50 mM Tris-HCl pH 7.5, 150 mM NaCl, 0.1 % sodium dodecyl sulfate (SDS), 0.5 % sodium deoxycholate (SDC), 1 % Triton X-100, with protease inhibitor cocktail (Merck, USA)). The lysates were left on ice for 10 min, and centrifuged at 16,000 G, at 4 °C for 10 min. The supernatants containing proteins were used for streptavidin affinity purification.

Bulk streptavidin affinity purification: To enrich biotinylated proteins from the lysates, streptavidin magnetic beads (Thermo Fisher Scientific) were used. Streptavidin magnetic beads, 30 µL, were first washed with 1 mL RIPA buffer, then 1 mL cell lysate was added to the beads and incubated at room temperature for 1 h with gentle shaking. The beads were then washed twice with 1 mL of RIPA buffer, once with 1 mL of 1 M KCl, 1 mL of 0.1 M sodium carbonate (Na_2CO_3), 1 mL of 2 M urea in 20 mM Tris-HCl pH 8, and finally twice with 1 mL 50 mM ammonium bicarbonate (ABC). Then, 200 µL of 50 mM ABC was added to the beads, and reduction of cysteine residues was performed with 10 mM DTT at room temperature for 30 min, followed by alkylation with 50 mM IAA at room temperature for 30 min. The proteins on beads were digested with 0.5 µg trypsin (Promega) at 37 °C overnight. Afterwards, the peptides in solution were separated from the beads using a magnetic rack and the peptides were desalted using SDB-XC StageTips [2].

1.7 TurboID reaction *in vitro* (related to Figure 2)

Biotinylation: 24 h after transfection with the MFG-E8 D49E-mCherry-TurboID plasmid into HEK293T cells in two 15 cm plates per condition, the medium was replaced with FBS-free DMEM, and the cells were incubated for another 2 days. SEVs were collected according to the protocol described in 1.4 Ultracentrifugation for SEV isolation, and TurboID-EV pellets were resuspended in 1 mL of PBS. Afterwards, 50 µM Biotin and 1 mM ATP (adenosine 5'-triphosphate disodium salt trihydrate (Fujifilm Wako, Japan) dissolved in 1 M Tris-HCl pH 7.5) was added to the supernatant, and incubated at 37 °C for 4 h.

Acetone precipitation

To remove free biotin and to precipitate proteins, ice-cold acetone was added to the suspension to a final concentration of 80 %, and incubated at −20 °C for 1 h and centrifuged at 20,000 G for 10 min at 4 °C. The supernatant was removed, and the pellet was washed

carefully once using cold acetone. Evaporate the residual acetone with a centrifugal vacuum concentrator, and the pellets were suspended with 100 µL of the RIPA buffer.

Spintip-based streptavidin affinity purification: We slightly modified the original protocol [3] for the purification of biotinylated proteins with streptavidin sepharose (Cytiva, USA). Two plugs of the C18 solid phase extraction (SPE) discs (GL Sciences, Japan) were inserted into a 200 µL pipette tip. For conditioning, 60 µL of MeOH was added and centrifuged at 1,000 G for 1 min. For blocking C18, 60 µL of 2 % SDS was added and centrifuged at 1,500 G for 1 min. Then, 1 µL of streptavidin sepharose slurry was loaded and centrifuged at 600 G for 1 sec. EV lysate was then loaded onto the StageTip and centrifuged at 100 G for 1 h. After biotinylated proteins were captured, the tips were washed 4 times with 60 µL of RIPA buffer at 1,500 G for 1 min. Then C18 was activated with 60 µL of 0.5 % (v/v) acetic acid (HOAc) in 80 % ACN by centrifugation at 1,500 G for 1 min. For reduction of cysteine residues, 20 µL of 50 mM ABC containing 10 mM DTT was added and incubated at room temperature for 15 min, and then the solution was removed with centrifugation at 1,000 G for 30 sec. Digestion buffer, 2 µL, containing 0.25 µg/µL trypsin (Promega), 50 mM IAA, and 50 mM ABC was loaded onto the tips and the samples were incubated at 37 °C for 1 h to digest and alkylate the proteins. The digested peptides (retained on C18) were washed with 60 µL of 1 % formic acid at 1500 G for 1 min. Afterwards, the peptides were eluted with 60 µL of 0.5 % (v/v) HOAc in 80 % ACN at 200 G for 5 min. Peptides were further purified by C18-SCX and SDB-XC StageTips, as described previously [4] [2].

1.8 TurboID reaction in recipient cells (related to Figure 3)

EV uptake: To prepare TurboID-EVs, HEK293T cells were cultured in twelve 15 cm plates containing DMEM with 10 % FBS. Transfection with MFG-E8 D49E-mCherry-TurboID plasmid DNA was performed at 80 % cell density. Next day, the medium was replaced with FBS-free DMEM, and the cells were incubated for another few days. SEVs corresponding to 2×10^{10} particles were collected using ultracentrifugation according to the protocol above, and TurboID-EV pellets were resuspended in DMEM, containing SILAC heavy amino acids, and 10 % SEV-depleted FBS, and added to recipient HEK293T cells labeled with SILAC heavy amino acids. Biotin was also added to the medium at 50 µM, and the cells were incubated for 4 h at 37 °C.

Fluorescence Activated Cell Sorting (FACS): After incubation with TurboID-EVs and biotin for 4 hours, cells were collected in PBS to 2.0×10^6 cells/mL. mCherry-positive and mCherry-negative cells were sorted using a BD FACS AriaII (BD, USA). After sorting, the collected cells were lysed and biotinylated proteins were enriched using C18-streptavidin sepharose spintips as described in Spintip-based streptavidin affinity purification.

1.9 Transmission electron microscopy (related to Figure 1B)

EV samples collected by centrifugation were placed on a carbon-coated grid for 10 min, followed by negative staining for one minute after washing off the sample solution with 2 % uranium acetate solution. Staining was removed with filter paper and the grid was dried. EV samples were viewed using an H-7650 transmission electron microscope (Hitachi, Japan). Digital images were captured using XR-41C CCD camera system (Advanced Microscopy Techniques, USA).

1.10 Nanoparticle tracking analysis (related to Figure 1B)

The concentration and size distribution of EVs were measured based on Brownian motion using a NanoSight NS300 nanoparticle characterization instrument (Malvern Panalytical, Japan). For each acquisition, five shots of 60 s each were used. The average of the five captures for each biological replicate was used to determine the mode of size distribution and nanoparticle concentration.

1.11 Immunoblotting (related to Figure S1B)

LDS (lithium dodecyl sulfate, pH 8.4) buffer (Thermo Fisher Scientific) containing 50 mM DTT was added to cell lysates in the RIPA buffer, and heated at 70 °C for 5 min. The samples were loaded onto an SDS–polyacrylamide gel electrophoresis (SDS-PAGE) gel (NuPAGE™ 4 to 12 %, Bis-Tris Gel, Thermo Fisher Scientific). After SDS-PAGE, proteins were then transferred to a polyvinylidene fluoride (PVDF) membrane (Merck) and stained with Ponceau (Beacle, Japan) to visualize total protein levels. BSA, 3 % (w/w), in tris-buffered saline (TBS)-Tween was used for blocking and inverted and mixed overnight. The membrane was then reacted with streptavidin-HRP at a 10,000:1 dilution for 1 h. After four washes with TBS-Tween, the membrane was reacted with ECL reagent (Merck).

1.12 Zeta potential measurement (related to Figure 1C)

Zeta potential measurements were carried out in 10 mM PBS at 25 °C using a Zetasizer Nano ZSP instrument (Malvern Instruments, UK). Three independent experiments were performed. SEVs from wild-type and TurboID-expressing HEK293T cells were diluted to a concentration of 1 µg protein/mL for zeta potential measurements.

1.13 LC/MS/MS analysis

In all experiments except for Figure 1D, proteome analyses were performed on an UltiMate 3000 RSLCnano system (Thermo Fisher Scientific), coupled with an Orbitrap Exploris 480 mass spectrometer (Thermo Fisher Scientific). All mass spectrometric analyses were carried out in the data-dependent acquisition (DDA) mode. Peptides were separated on self-pulled needle columns[5] (250 mm, 100 µm ID) packed with Reprosil-Pur 120 C18-AQ 1.9 µm (Dr. Maisch, Ammerbuch, Germany) at 50 °C in a column oven (Sonation GmbH, Germany). Mobile phase A and B were 0.5 % acetic acid and 0.5 % acetic acid in 80 % ACN, respectively. The flow rate was 400 nL/min. The flow gradient was set as follows: 5 % B in 5 min, 5–19 % B in 55.3 min, 19–29 % B in 21 min, 29–40 % B in 8.7 min, and 40–99 % B in 0.1 min, followed by 99 % B for 4.9 min. The electrospray voltage was set to 2.4 kV in the positive mode. The mass spectrometric analysis was carried out with the FAIMS Pro interface. The FAIMS mode was set to a standard resolution, and the total carrier gas flow was 4.0 L/min. The CV was set to −40 and −60, and the cycle time of each CV experiment was set to 1 s. The mass range of the survey scan was from 375 to 1,500 *m/z* with a resolution of 60,000, 300 % normalized automatic gain control (AGC) target, and auto maximum injection time. The first mass of the MS/MS scan was set to 120 *m/z* with a resolution of 15,000, standard AGC, and auto maximum injection time. Fragmentation was performed by HCD with a normalized collision energy of 30 %. The dynamic exclusion time was set to 20 s.

Regarding the experiment in Figure 1D, proteome analyses were performed on an Ultimate3000 RSLCnano (Thermo Fisher Scientific), coupled with an Orbitrap Fusion Lumos mass spectrometer (Thermo Fisher Scientific) and HTC-PAL (CTC Analytics). All mass spectrometric analyses were carried out in the DDA mode. Peptides were separated on self-pulled needle columns[5] (250 mm, 100 µm ID) packed with Reprosil-Pur 120 C18-AQ 3.0 µm

(Dr. Maisch, Ammerbuch, Germany). The flow rate was 500 nL/min. The flow gradient was set as follows: 5–10 % B in 5 min, 10–40 % B in 60 min, 40–99 % B in 5 min, followed by 99 % B for 10 min. The electrospray voltage was set to 2.4 kV in the positive mode. The mass spectrometric analysis was carried out with the FAIMS Pro Duo interface. The FAIMS mode was set to a standard resolution, and the total carrier gas flow was 4.6 L/min. The CV was set to −40, -60 and −80, and the cycle time of each CV experiment was set to 1 s. The mass range of the survey scan was from 300 to 1,500 *m/z* with a resolution of 120,000, standard AGC and 50 ms of maximum injection time. The MS/MS scan was performed on ion trap with rapid ion trap scan rate, standard AGC, and 35 ms of maximum injection time. Fragmentation was performed by HCD with a normalized collision energy of 30 %. The dynamic exclusion time was set to 20 s.

1.14 Proteomics data processing and analysis

All raw data files were analyzed and processed using MaxQuant (v1.6.17.0)[6], and the database search was performed with Andromeda [6,7] against the UniProt human fasta file (version 2019-3) spiked with common contaminants and enzyme sequences. Raw data files collected from FAIMS experiments were split into a set of MaxQuant compliant MzXML files using FAIMS MzXML Generator (https://github.com/coongroup/FAIMS-MzXML-Generator) [8]. Search parameters included two missed cleavage sites and variable modifications such as methionine oxidation, protein N-terminal acetylation, and for the SILAC experiment related to Figure 3, L-($^{13}C_6$,$^{15}N_4$)-arginine (Arg10), L-($^{13}C_6$,$^{15}N_2$)-lysine (Lys8). Cysteine carbamidomethylation was set as a fixed modification. The peptide mass tolerance was 4.5 ppm, and the MS/MS tolerance was 20 ppm. The false discovery rate (FDR) was set to 1 % at the peptide spectrum match (PSM) level and protein level. For protein-level quantification in all experiments, ‘unique + razor’ peptides were used. Proteins quantified from at least 1 unique peptide ions were used. For volcano plots related to Figures 1D and 2B, only proteins quantified in 2 out of the 3 replicates in at least one condition were used for further analysis, and missing values were imputed from a normal distribution of $\log_2$ intensity using a default setting (width 0.3, down shift 1.8) in Perseus (v.1.6.5.0) [9]. Volcano plots were generated based on $\log_2$ FC (x-axis) and $-\log_{10}$ p-value from two-sided t-test (y-axis). ExoCarta (http://www.exocarta.org/) was used to indicate whether corresponding proteins are EV-related or not in Tables S1-4.

2. Supplementary Figures

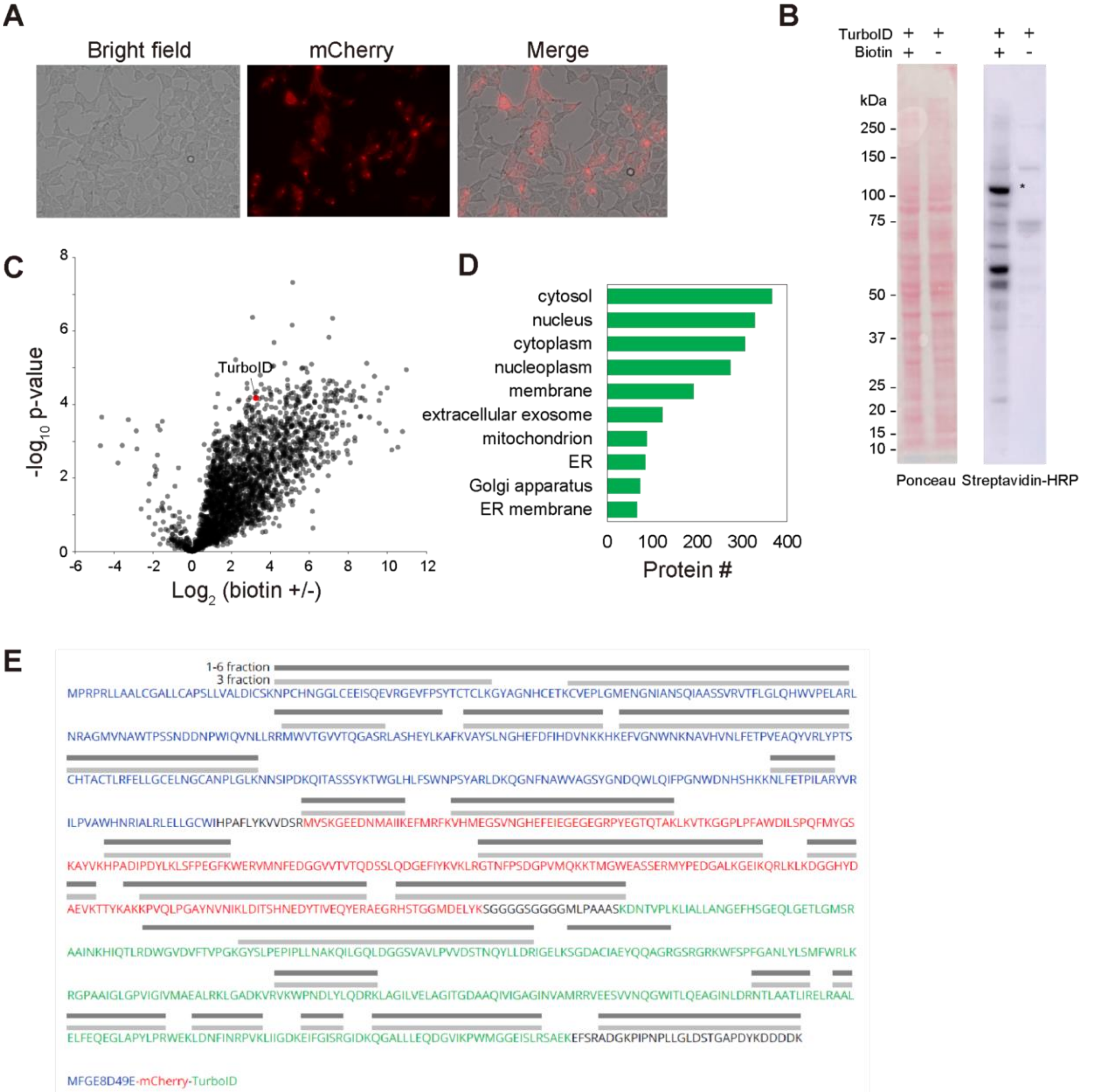

Figure S1: Mass spectrometric analysis of the TurboID fusion protein (related to Figure 1)

(A) Fluorescence microscopy images obtained 24 h after transfection of the TurboID plasmids into HEK293T cells. (B) Ponceau staining (left) and streptavidin-HRP blot (right) of whole-cell lysates of HEK293T cells transiently expressing the TurboID protein. * indicates the expected position of the 105 kDa TurboID fusion protein. (C) A volcano plot showing differential biotinylation levels of proteins in HEK293T cells expressing the TurboID proteins with or without biotin addition. Three independent experiments were performed. (D) Cellular components of proteins enriched in the biotin (+) experiments (log_2 fold-change> 2 and p<0.01). (E) Sequence coverage of the peptides derived from the TurboID fusion protein identified in Figure 1C.

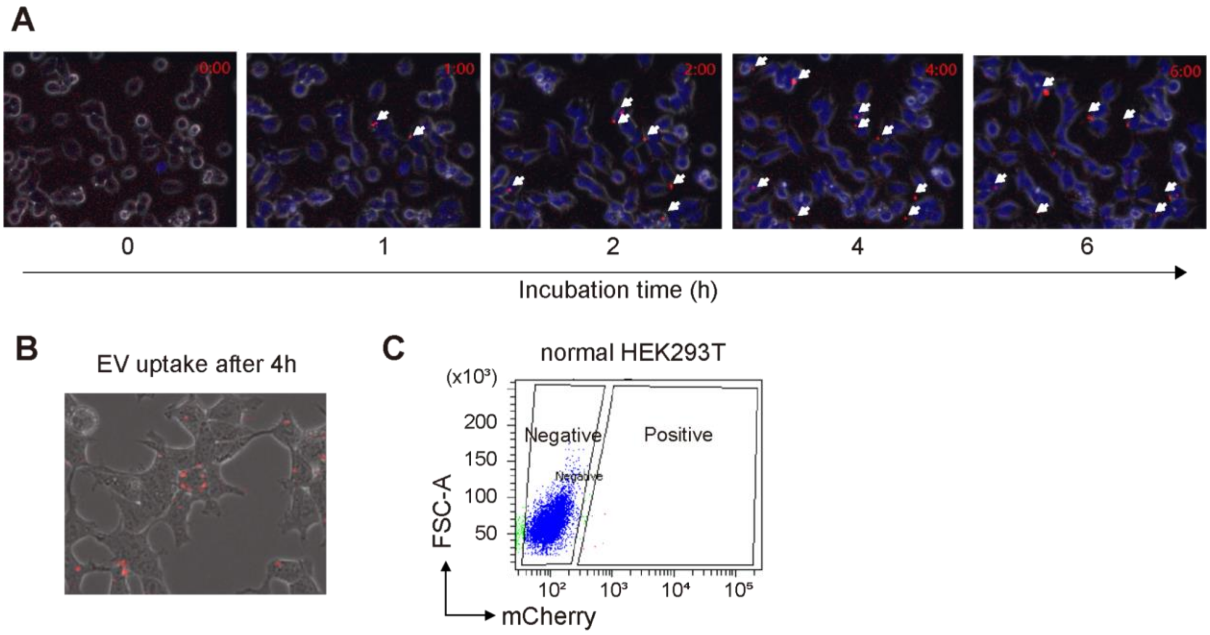

Figure S2: Evaluation of TurboID-EV uptake and FACS experiments (related to Figure 3A).

(A) Time-lapse images of EV uptake processes in HEK293T cells. The white arrows indicate MFGE8-mCherry-EVs taken up by HEK293T cells. Red and blue fluorescence represent EVs and nuclei stained with DAPI. (B) Fluorescence microscopy images obtained 4 h after the addition of TurboID-EVs and biotin to normal HEK293T cells (just before FACS sorting). (C) Flow cytometric analysis of EVs collected from normal HEK293T cells (that did not express the TurboID proteins) as a control.

3. Supplementary References

(1) Jeppesen, D. K.; Fenix, A. M.; Franklin, J. L.; Higginbotham, J. N.; Zhang, Q.; Zimmerman, L. J.; Liebler, D. C.; Ping, J.; Liu, Q.; Evans, R.; Fissell, W. H.; Patton, J. G.; Rome, L. H.; Burnette, D. T.; Coffey, R. J. Reassessment of Exosome Composition. *Cell* **2019**, *177* (2), 428–445.e18.

(2) Rappsilber, J.; Ishihama, Y.; Mann, M. Stop and Go Extraction Tips for Matrix-Assisted Laser Desorption/ionization, Nanoelectrospray, and LC/MS Sample Pretreatment in Proteomics. *Anal. Chem.* **2003**, *75* (3), 663–670.

(3) Mao, Y.; Chen, P.; Ke, M.; Chen, X.; Ji, S.; Chen, W.; Tian, R. Fully Integrated and Multiplexed Sample Preparation Technology for Sensitive Interactome Profiling. *Anal. Chem.* **2021**, *93* (5), 3026–3034.

(4) Adachi, J.; Hashiguchi, K.; Nagano, M.; Sato, M.; Sato, A.; Fukamizu, K.; Ishihama, Y.; Tomonaga, T. Improved Proteome and Phosphoproteome Analysis on a Cation Exchanger by a Combined Acid and Salt Gradient. *Anal. Chem.* **2016**, *88* (16), 7899–7903.

(5) Ishihama, Y.; Rappsilber, J.; Andersen, J. S.; Mann, M. Microcolumns with Self-Assembled Particle Frits for Proteomics. *J. Chromatogr. A* **2002**, *979* (1-2), 233–239.

(6) Cox, J.; Mann, M. MaxQuant Enables High Peptide Identification Rates, Individualized P.p.b.-Range Mass Accuracies and Proteome-Wide Protein Quantification. *Nat. Biotechnol.* **2008**, *26* (12), 1367–1372.

(7) Cox, J.; Neuhauser, N.; Michalski, A.; Scheltema, R. A.; Olsen, J. V.; Mann, M. Andromeda: A Peptide Search Engine Integrated into the MaxQuant Environment. *J. Proteome Res.* **2011**, *10* (4), 1794–1805.

(8) Hebert, A. S.; Prasad, S.; Belford, M. W.; Bailey, D. J.; McAlister, G. C.; Abbatiello, S. E.; Huguet, R.; Wouters, E. R.; Dunyach, J.-J.; Brademan, D. R.; Westphall, M. S.; Coon, J. J. Comprehensive Single-Shot Proteomics with FAIMS on a Hybrid Orbitrap Mass Spectrometer. *Anal. Chem.* **2018**, *90* (15), 9529–9537.

(9) Tyanova, S.; Temu, T.; Sinitcyn, P.; Carlson, A.; Hein, M. Y.; Geiger, T.; Mann, M.; Cox, J. The Perseus Computational Platform for Comprehensive Analysis of (prote)omics Data. *Nat. Methods* **2016**, *13* (9), 731–740.
